# A Novel Aptamer Selection Strategy for *Pseudomonas aeruginosa* and Its Application as a Detecting Probe in a Hybrid Lateral Flow Assay

**DOI:** 10.3390/molecules30173499

**Published:** 2025-08-26

**Authors:** Thu Thao Pham, Nguyen T. T. Huyen, Le Hong Oanh, Lam Dai Tran, Hiep V. Tran, T. N. Lien Truong, Nguyen Thi Phuong Trang

**Affiliations:** 1 Vietnam Academy of Science and Technology, Graduate University of Science and Technology, Hanoi 100000, Vietnam; phamthao.tpr@gmail.com; 2 Vietnam-Korea Institute of Science and Technology, Hoa Lac Hi-Tech Park, Hanoi 100000, Vietnam; nguyenhuyen@mic.gov.vn (N.T.T.H.); lhoanh@mic.gov.vn (L.H.O.); ttnlien@mic.gov.vn (T.N.L.T.); 3 Institute of Tropical Technology, Vietnam Academy of Science and Technology, 18 Hoang Quoc Viet Road, Hanoi 100000, Vietnam; lamtd@ims.vast.ac.vn

**Keywords:** *Pseudomonas aeruginosa*, aptamer, whole-cell SELEX, lateral flow immunoassay, gold nanoparticles, hybrid biosensor

## Abstract

*Pseudomonas aeruginosa* is a clinically significant pathogen with high antibiotic resistance, necessitating rapid and reliable diagnostic methods. In this study, we developed a whole-cell aptamer selection method for *P. aeruginosa* using an Eppendorf-tube-based SELEX system, where bacterial cells were directly incubated with an ssDNA library. This configuration enhanced the recovery of bound aptamers and overcame the cell quantity limitations often encountered in microtiter-plate-based SELEX. After 10 selection rounds, six aptamer candidates were obtained and evaluated for affinity. Molecular docking analysis revealed that aptamer T1 possessed the highest target selectivity. To demonstrate diagnostic applicability, aptamer T1 was integrated into a hybrid lateral flow immunoassay (LFIA), replacing the conventional detection antibody. In this format, the AuNP–aptamer complex bound to the target bacteria and was captured by a specific antibody immobilized on the test line. The LFIA achieved a visual detection limit of 2.34 × 10^2^ CFU/mL within 15 min, showing high specificity and suitability for point-of-care applications. This study presents the first demonstration of an aptamer–antibody hybrid LFIA for bacterial detection and highlights the potential of aptamers as low-cost, rapidly synthesized recognition elements adaptable for the detection of other infectious agents.

## 1. Introduction

*Pseudomonas aeruginosa* is a Gram-negative, rod-shaped, motile bacterium commonly found in natural environments such as soil and water, as well as in artificial settings including hospitals [1]. Among the *Pseudomonas* genus, *P. aeruginosa* is the most clinically significant species due to its role as an opportunistic pathogen, particularly affecting immune-compromised individuals such as patients with HIV/AIDS, cancer, or severe burns. It is associated with a wide range of life-threatening infections, including bacteremia, pneumonia, meningitis, and urinary tract infections. Its ability to survive under aerobic, anaerobic, or low-oxygen conditions and its inherent resistance to multiple antibiotics make it especially challenging to treat and control [2].

Traditional diagnostic techniques for *P. aeruginosa* detection (such as PCR, ELISA, etc.) are often time-consuming, labor-intensive, and require expensive instrumentation, limiting their application in low-resource settings. Therefore, the development of a rapid, sensitive, and cost-effective detection method is urgently needed for clinical diagnostics and infection control. Aptamers are short, single-stranded nucleic acids that can bind specifically and with high affinity to a wide range of targets, including small molecules, proteins, whole cells, and pathogens. Over the past two decades, aptamer-based detection platforms have attracted considerable interest as alternatives to antibody-based assays due to their chemical stability, cost-effectiveness, and ease of large-scale synthesis with high batch-to-batch reproducibility [3,4]. In the context of bacterial and viral diagnostics, most reported selection strategies have relied on purified antigens or recombinant proteins as targets, enabling aptamer generation against specific epitopes. While effective, these approaches require prior knowledge of the antigen and its production in a purified form, which can be time-consuming and may fail to capture structural or conformational determinants present on intact pathogens. In recent years, whole-cell SELEX has emerged as a promising alternative, enabling the selection of aptamers directly against intact bacterial or viral particles without the need for antigen purification [5,6]. This strategy offers two key advantages: (i) it allows aptamer development to proceed as soon as the pathogen is available, bypassing the antigen production bottleneck; and (ii) it can yield a diverse repertoire of aptamers recognizing multiple surface epitopes, potentially improving detection robustness and specificity. Whole-cell SELEX has been successfully applied to various bacterial and viral species, demonstrating its potential for broad diagnostic applications.

This study developed a whole-cell aptamer selection method for *Pseudomonas aeruginosa* using an Eppendorf-tube-based system in which bacterial cells were incubated directly with an ssDNA library. This configuration not only increased the recovery of bound aptamers but also removed limitations on cell quantity often encountered in microtiter-plate-based SELEX. The selected aptamers were subsequently integrated into a lateral flow immunoassay (LFIA) platform, replacing conventional antibodies.

Our aptamer-based LFIA enables visual detection of *P. aeruginosa* within 15 min with a detection limit of 10^2^ CFU/mL. This integration leverages the inherent advantages of aptamers—lower production costs, full synthetic accessibility, and batch homogeneity—while enabling rapid, on site detection of the target pathogen in clinical specimens. The methodological improvements presented here can be readily adapted for aptamer generation against a broad range of bacterial and viral targets, extending the applicability of LFIA-based diagnostics beyond *P. aeruginosa*.

The outcomes of this study have been legally protected under Patent Application No. 2-2025-00350.

## 2. Materials and Methods

### 2.1. Materials and Reagents

All chemicals and biological reagents used in this study were of analytical grade. Taq PCR Master Mix (2×), GeneRuler 100 bp DNA Ladder (M100), GeneRuler 25 bp DNA Ladder (M25), and 6× DNA loading dye were purchased from Thermo Scientific (Waltham, MA, USA). RedSafe Nucleic Acid Staining Solution was obtained from INtRON Biotechnology (Seongnam-si, Republic of Korea). Agarose, 10× TBE buffer, and Tween-20 were acquired from Sigma-Aldrich (Saint Louis, MO, USA). The Quick Gel Extraction Kit was obtained from Invitrogen (Carlsbad, CA, USA).

The initial single-stranded DNA (ssDNA) library and primers were synthesized by Integrated DNA Technologies (IDT, Coralville, IA, USA). The sequence of the forward primer (Aptamer F-primer) was 5′-ATC CGT CAC ACC TGC TCT-3′, and the reverse primer (Aptamer R-primer) was 5′-ATA CGG GAG CCA ACA CCA-3′.

Inactivated bacterial strains of *Pseudomonas aeruginosa* (VTCC 12273) and *Klebsiella pneumoniae* (VTCC 12018) were kindly provided by the Institute of Microbiology and Biotechnology, Vietnam National University, Hanoi, Vietnam.

Mouse IgG antibodies used for comparative assays were purchased from Invitrogen (USA). Materials for lateral flow assay (LFA) strip fabrication—including nitrocellulose membranes, sample pads, conjugate pads, and absorbent pads—were obtained from Merck (Darmstadt, Germany).

For cloning experiments, the TOPO TA Cloning^®^ Kit and chemically competent *Escherichia coli* DH5α cells were purchased from Invitrogen (USA).

### 2.2. Methods

#### 2.2.1. SELEX Procedure

Preparation and Denaturation of Aptamer Library: A synthetic ssDNA library consisting of 91 nucleotides (5′-ATCCGTCACACCTGCTCT–(N)_55_–TGGTGTTGGCTCCCGTAT-3′) was used, with a 55-nucleotide randomized central region flanked by fixed primer-binding sites. The library was diluted in 1× PBS to 100 μM, denatured at 95 °C for 5 min, and rapidly cooled on ice for 10 min to enable proper folding [7].

##### Target Binding and Washing

Fifty microliters of the folded ssDNA library were incubated with 100 μL of heat-inactivated *P. aeruginosa* (10^8^ CFU/mL) at room temperature for 180 min under gentle agitation. After incubation, cells were pelleted by centrifugation (6000 rpm, 5 min) and washed 3–5 times with 1X PBS ontaining 0.7% Tween-20. A final rinse was performed with sterile distilled water [8,9].

##### Elution and Amplification

Bound aptamers were eluted by heating at 95 °C for 10 min, followed by centrifugation (12,000 rpm, 5 min). The supernatant containing aptamers was used as a template for PCR amplification using the following primers:

Forward: 5′-ATC CGT CAC ACC TGC TCT-3′

Reverse: 5′-ATA CGG GAG CCA ACA CCA-3′

PCR was carried out with an initial denaturation at 95 °C for 3 min, followed by 10 cycles of 95 °C for 30 s, 56 °C for 30 s, 72 °C for 30 s, and a final extension at 72 °C for 5 min. PCR products were verified via 2.5% agarose gel electrophoresis.

##### Asymmetric PCR and Iterative Selection

Asymmetric PCR (A-PCR) was performed to generate ssDNA for subsequent SELEX rounds. A total of 10 SELEX rounds were conducted, with increasing selection pressure achieved by gradually reducing incubation time (the incubation time in each successive round was reduced to two-thirds of that in the previous round, while the concentrations of both the bacterial cells and the ssDNA library were kept constant).

Counter-selection was conducted in rounds 5 and 7 using *E. coli* and *K. pneumoniae* to eliminate non-specific aptamers [4,9].

#### 2.2.2. Cloning and Sequence Analysis

After the final round, aptamer pools were cloned into the TOPO TA vector and transformed into chemically competent *E. coli* DH5α cells. Colonies were screened on LB agar containing kanamycin and X-gal. A total of 16 positive colonies were identified via colony PCR using universal M13 primers and sequenced by the Sanger method. Six unique aptamer sequences were selected.

#### 2.2.3. Docking and Structure Modeling

The secondary structures of selected aptamers were predicted using RNAfold. Tertiary structures were modeled by RNAComposer. The 3D model of the *P. aeruginosa* lipopolysaccharide (LPS) was constructed using the Glycam web server. The binding interactions between the aptamers and LPS (docking) were subsequently simulated using the HDOCK platform [10,11,12].

#### 2.2.4. Synthesis and Characterization of AuNPs

**Figure 1:**
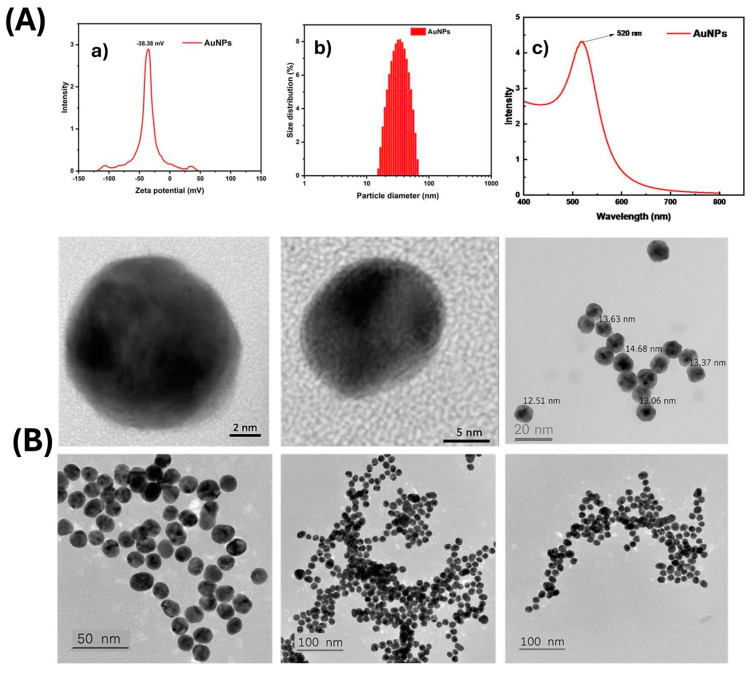
The physico-chemical characteristics of the synthesized AuNPs. (**A**) Optical characteristics and size distribution of gold nanoparticles (AuNPs): (**a**) zeta potential distribution; (**b**) particle size distribution determined by dynamic light scattering (DLS); (**c**) UV–Vis absorption spectrum of AuNPs. (**B**) High-resolution transmission electron microscopy (HR-TEM) images illustrating the morphology and size distribution of the AuNPs.

Spherical AuNPs (12–15 nm) were synthesized via citrate reduction [13,14]. Gold nanoparticles (AuNPs) were comprehensively characterized in terms of their physico-chemical properties—the zeta potential of the AuNPs was measured to be approximately –38.38 mV, indicating high colloidal stability due to strong electrostatic repulsion among negatively charged particles. Figure 1(Aa,Ab) presents the particle size distribution obtained via dynamic light scattering (DLS), demonstrating a narrow and uniform size distribution, which confirms good dispersibility of the synthesized nanoparticles. Figure 1(Ac) displays the UV–Vis absorption spectrum, revealing a characteristic surface plasmon resonance (SPR) peak at around 520 nm, consistent with spherical AuNPs of approximately 15–20 nm in diameter. High-resolution TEM images clearly show nearly spherical shapes and well-defined crystalline structures of individual particles. The average particle size was found to be in the range of 12–15 nm, in good agreement with the DLS results. Lower-magnification TEM images revealed well-dispersed particles with a high degree of monodispersity and no obvious aggregation, confirming the stability and high quality of the synthesized AuNPs (Figure 1B). The gold nanoparticle synthesis protocol was registered for patent (Application No.: 2-2024-00075).

#### 2.2.5. Fabrication of LFIA

##### Aptamer–AuNP Conjugation

A mixture of 1 vol of AuNPs and 0.06 vol of 2.8 mg/mL aptamer solution was incubated at 60 °C for 1 h to allow for electrostatic adsorption.

##### IgG Antibody–AuNP Conjugation

AuNPs were modified with 0.1 mM MUA (Mercaptosuccinic acid) and activated with 0.1 M NHS and 0.2 M EDC. Goat anti-mouse IgG (1 mg/mL) was added and incubated for 1h at room temperature. BSA was used for blocking non-specific sites.

##### Assembly and Processing of Test Strip Components

**Figure 2:**
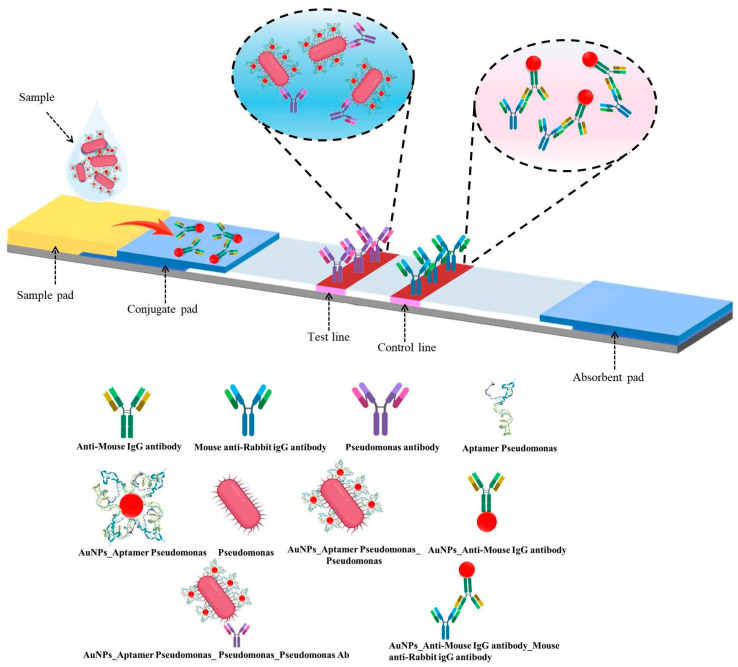
Schematic representation of the LFIA strip: structural components and detection mechanism.

The LFIA strip consisted of a sample pad, conjugate pad, nitrocellulose (NC) membrane, and absorbent pad [15]. Pads were pretreated with 10 mM HEPES buffer (pH 7.4) containing 1% BSA, 1% sucrose, 0.5% Tween-20, and 0.05% sodium azide. The NC membrane was coated with polyclonal rabbit anti-*P. aeruginosa* antibodies (1 mg/mL) on the test line (T) and anti-mouse IgG on the control line (C). AuNP–IgG conjugates were sprayed onto the conjugate pad. After drying, all components were assembled, and strips were cut into 3-millimeter widths. In the test strip design, the conjugate pad was pre-loaded with AuNP–mouse IgG complexes, serving as the internal control signal. The test line (T) was immobilized with an antibody specific to *P. aeruginosa* (capture antibody), enabling the capture of the AuNP–aptamer–bacterium complex via bacterial surface antigens. The control line (C) was coated with anti-mouse IgG antibodies to capture the AuNP–mouse IgG complex from the conjugate pad. Upon sample application to the sample pad, the mixture migrated along the nitrocellulose membrane by capillary action. In the presence of the target bacterium, the AuNP–aptamer–bacterium complex was retained at the test line, resulting in the appearance of a red band at this location. Simultaneously, the AuNP–mouse IgG control conjugate interacted with the anti-mouse IgG at the control line, generating a red band that confirmed the proper functioning of the assay (Figure 2).

#### 2.2.6. Evaluation of Colloidal Stability of AuNP–Aptamer Conjugates via Salt-Induced Aggregation

To indirectly evaluate the surface coverage and colloidal stability of the AuNP–aptamer conjugates, a salt-induced aggregation assay was performed. After the aptamer conjugation process was completed, 10 µL of 10% NaCl solution was added to 100 µL of the conjugate solution and gently mixed. If the AuNPs were not fully covered by aptamers, the salt would reduce the electrostatic repulsion between particles, leading to aggregation and a color change from red to purple or blue. In contrast, retention of the characteristic red color indicated successful aptamer attachment and high colloidal stability of the AuNP–aptamer conjugates.

#### 2.2.7. Sample Detection Procedure

Samples containing *P. aeruginosa* at concentrations ranging from 10^1^ to 10^9^ CFU/mL were mixed with AuNP–aptamer conjugates at a 1:0.03 (*v*/*v*) ratio and incubated at 60 °C for 30 min. The resulting mixtures were applied to the sample pad of the LFIA strip. In positive samples, a red band appeared at the test line due to accumulation of AuNP–aptamer–bacteria complexes, while a red band at the control line confirmed assay validity. Negative controls included bacteria-free samples (blank) and samples containing *Escherichia coli* or *Klebsiella pneumonia* (10^3^ CFU/mL), which did not produce a visible test line.

## 3. Results and Discussion

### 3.1. Aptamer Enrichment and Characterization

**Figure 3:**
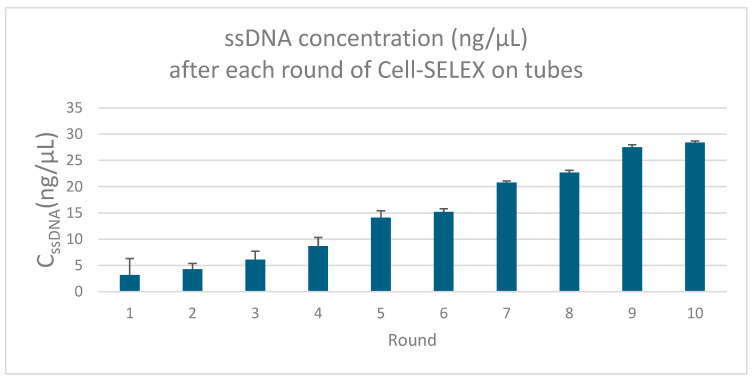
Illustration of the ssDNA concentrations across SELEX rounds (ssDNA concentrations obtained after each round of whole-cell SELEX against *P. aeruginosa*, measured by nanodrop spectrophotometry—the gradual increase indicates progressive enrichment of high-affinity sequences).

The concentration of single-stranded DNA (ssDNA) recovered after each round of SELEX increased gradually, indicating an enrichment of sequences with higher binding affinity to *P. aeruginosa* (Figure 3).

After ten rounds of whole-cell SELEX and counter-selection with *E. coli* and *K. pneumoniae* to eliminate non-specific binders, the enriched aptamer pool was cloned using the TOPO TA Cloning^®^ Kit. Sixteen white colonies were selected and subjected to plasmid extraction and Sanger sequencing using universal M13 primers. Sequence analysis revealed that six aptamer sequences were predominant, suggesting a convergence towards specific binding motifs. These six representative sequences were further analyzed for secondary structure (via Mfold) [11,16] and molecular docking affinity (via HDOCK) [13,17] with the lipopolysaccharide (LPS) region on the surface of *P. aeruginosa*. The lipopolysaccharide (LPS) region on the surface of *P. aeruginosa*, specifically the Common Polysaccharide Antigen (CPA) located at the distal end of the LPS molecule, serves as the primary binding target for the aptamers; this is because the *P. aeruginosa* strain VTCC 12273 (also known as ATCC 27853) expresses CPA but lacks the O-specific antigen (OSA) due to the absence of several genes required for OSA biosynthesis [18]. The absence of OSA renders CPA virtually the sole accessible epitope for aptamer recognition, thereby facilitating the selection of CPA-specific aptamers.

Details of the aptamer selection process—including docking scores, 2D/3D structure models, and docking interaction images—are provided in Table 1.

MFE (minimum free energy (ΔG)) refers to the lowest predicted free energy value. The MFE model represents a two-dimensional (2D) secondary structure of the aptamer, generated by maximizing the number of complementary base pairings to achieve the minimum free energy.

Frequency indicates the percentage of a particular aptamer conformation present in the environment. For instance, the MFE conformation of aptamer T1 appears with a frequency of 24.98%, meaning that approximately 25 out of every 100 aptamer molecules adopt this structure at any given time.

Diversity score is calculated based on the entropy distribution of each aptamer sequence. Higher entropy values reflect greater structural instability and, consequently, higher diversity. This score represents the overall structural variability in all predicted conformations associated with a single aptamer sequence.

Docking score represents the binding energy loss when the aptamer interacts with the LPS molecule [19,20,21,22].

Among the six selected aptamers, T1 was the most frequently identified sequence (found in 4 of 16 colonies), displaying the highest MFE conformation frequency, the lowest diversity score, and the most favorable docking score with *P. aeruginosa* CPA. Predominantly forming classical stem–loop motifs, T1 maintained its native structure upon antigen binding, while other aptamers exhibited marked structural distortion and antigen displacement. These attributes established T1 as the most promising candidate for subsequent evaluation in gold nanoparticle conjugation and *P. aeruginosa* detection.

**Figure 4:**
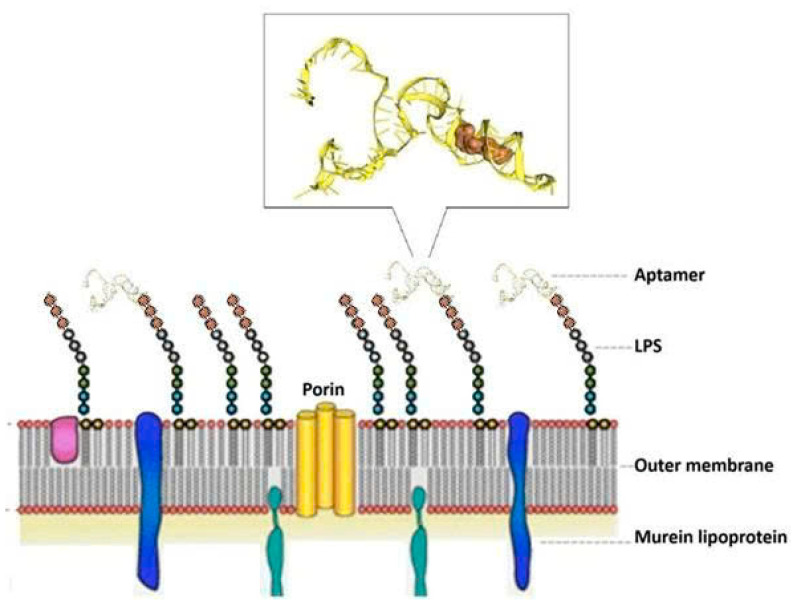
Docking interaction of aptamer T1 with CPA (of the LPS) target on the surface of *P. aeruginosa*.

The docking interaction models of aptamer T1 with the CPA (of the LPS) target are shown in Figure 4.

### 3.2. Aptamer T1–AuNP Conjugation and Optimization

To optimize the conjugation of aptamer T1 onto AuNPs, we investigated two key parameters that simultaneously affect the conjugation efficiency: temperature and aptamer concentration. For clarity, the effect of temperature was first evaluated at a fixed aptamer concentration to determine the optimal conjugation temperature. Subsequently, using this optimal temperature, the influence of aptamer concentration on conjugation efficiency was examined. This stepwise approach allows a systematic assessment of each parameter and facilitates the identification of conditions that yield the highest binding performance.

**Figure 5:**
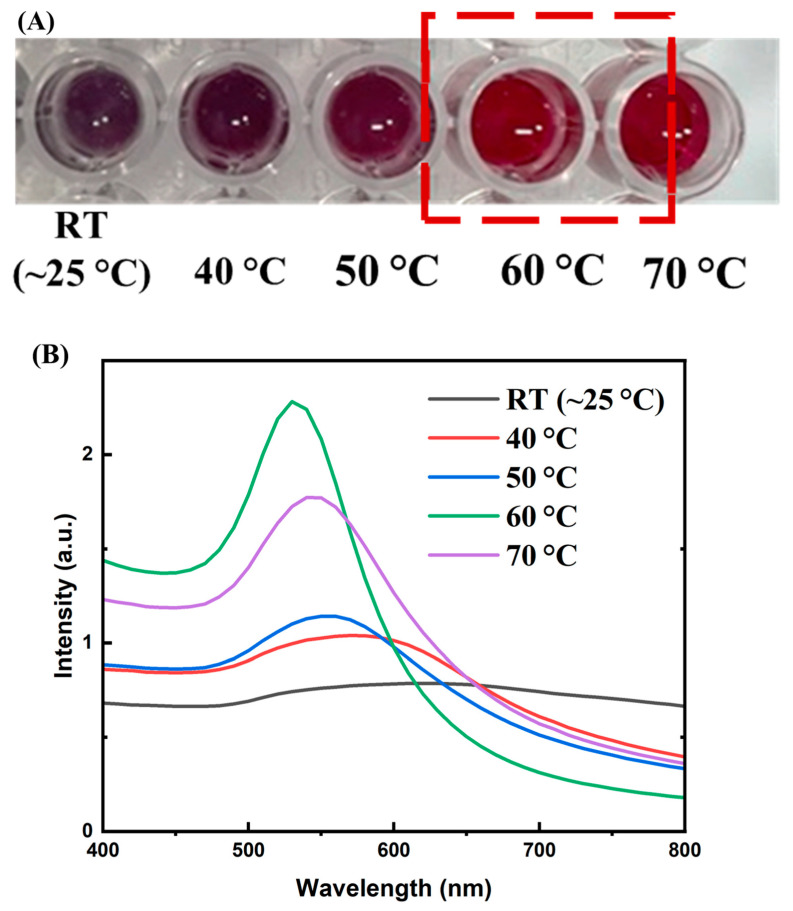
(**A**) Color of AuNP solutions incubated with aptamer T1 (0.80 mg/mL) at different temperatures (RT ~25 °C, 40 °C, 50 °C, 60 °C, and 70 °C) for 1 h after the addition of 10% NaCl. The RT sample turned deep purple, the 40 °C and 50 °C samples displayed lighter purple shades, whereas the 60 °C and 70 °C samples retained the characteristic bright red color of AuNPs; (**B**) UV–Vis spectra of the solutions in (**A**), showing the highest absorption intensity for the sample incubated at 60 °C, indicating the best colloidal stability of aptamer-conjugated AuNPs under this condition.

The conjugation of aptamers onto the surface of gold nanoparticles (AuNPs) via electrostatic interactions is often carried out at elevated temperatures to enhance the efficiency of binding and the stability of the aptamer T1 coating on the particle surface. At room temperature, aptamers—being oligonucleotide chains—tend to form secondary structures such as hairpins or stem–loops, which can shield the negatively charged phosphate groups and reduce their ability to interact with the gold surface. Increasing the temperature to approximately 50–70 °C temporarily disrupts these secondary structures, allowing the aptamer strand to unfold and expose its negatively charged regions, thereby enhancing electrostatic adsorption onto the AuNP surface [23]. Moreover, higher temperatures increase molecular kinetic energy, enabling aptamers to diffuse more rapidly in solution and more effectively approach and bind to the gold surface [24]. This process also helps prevent AuNP aggregation in solution—a risk that occurs when aptamers are unevenly attached or form bridges between particles. Notably, when thiol-modified aptamers are used to form covalent Au–S bonds, heating in the range of 50–70 °C remains safe as it does not disrupt these bonds, while still benefiting from aptamer unfolding during the conjugation process [25]. To optimize the conjugation temperature of aptamers to AuNPs, we incubated aptamer T1 (with a concentration of 0.80 mg/mL) with AuNPs in borate buffer (pH 9) for 1 h at various temperatures: room temperature (RT), 40, 50, 60, and 70 °C. Subsequently, 10% NaCl was added, and the color of the solution in each well was visually observed. As shown in Figure 5A, at RT the solution turned deep purple; at 40 °C and 50 °C the purple color gradually faded; at 60 °C and 70 °C the solution retained the characteristic bright wine-red color of AuNPs. The UV–Vis spectra of the solutions after adding 10% NaCl (Figure 5B) showed the highest absorption intensity for the sample incubated at 60 °C. Thus, the colloidal stability of aptamer-conjugated AuNPs with heat-assisted binding was evaluated using visual colorimetric observation and UV–Vis spectrophotometry. Based on these results, we selected 60 °C as the optimal temperature for aptamer conjugation onto AuNPs.

**Figure 6:**
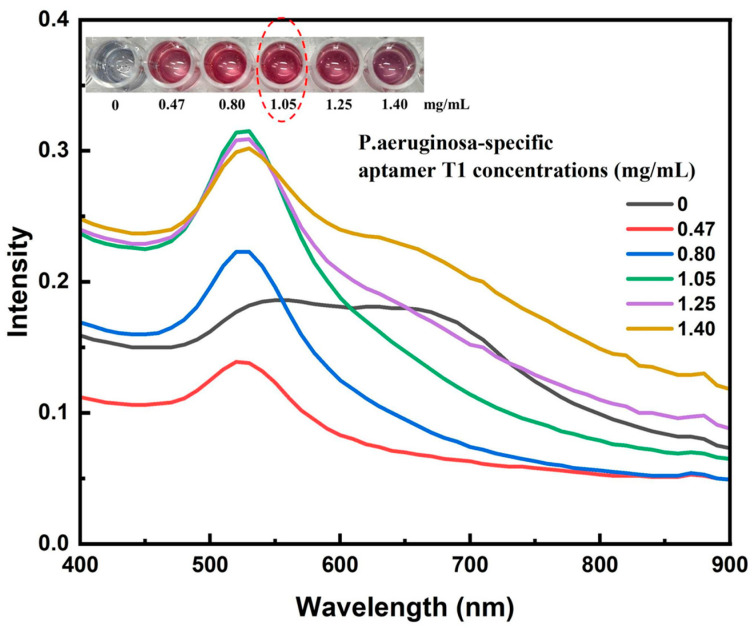
UV–Vis absorption spectra of AuNP–aptamer T1 conjugates prepared with varying concentrations of the *P. aeruginosa*-specific aptamer after NaCl addition, showing plasmon peak shifts associated with nanoparticle aggregation and stability.

Having established the optimal conjugation temperature, the subsequent experiment focused on evaluating how varying aptamer concentrations influence the conjugation efficiency under this fixed-temperature condition. The conjugation and stabilization capacity of the AuNP–aptamer complex were evaluated by incubating AuNPs with various volumes (0, 5, 10, 15, 20, and 25 µL) of an aptamer T1 stock solution (2.8 mg/mL), corresponding to final concentrations of 0, 0.47, 0.80, 1.05, 1.25, and 1.40 mg/mL, respectively. The mixtures were incubated in borate buffer (pH 9) at 60 °C for 1 h. Subsequently, 10 µL of 10% NaCl solution was added to each mixture to assess salt-induced aggregation—an indirect indicator of surface coverage and nanoparticle stability. Visual observations revealed that only at aptamer concentrations of 0.47, 1.25, and 1.40 mg/mL did the solution color shift from the characteristic ruby red of stable AuNPs to a pale purple upon salt addition (see inset of Figure 6). This color change indicates nanoparticle aggregation, which occurs when the aptamer coverage on the AuNP surface is not optimal to provide electrostatic and steric stabilization, allowing salt-induced charge screening to promote particle clustering. UV–Vis spectral analysis supported these observations, revealing a marked decrease in intensity or a red shift of the plasmon peak from ~520 nm at lower aptamer concentrations, indicative of nanoparticle aggregation. In contrast, at higher aptamer concentrations (≥1.05 mg/mL), the solution retained its characteristic ruby-red color, and the UV–Vis spectra displayed a sharp plasmon resonance peak between 520 and 525 nm, consistent with stable, non-aggregated AuNPs. Notably, further increasing the aptamer concentration beyond 1.05 mg/mL produced no substantial improvement in stability, suggesting that the AuNP surface had reached saturation coverage. Therefore, a final concentration of aptamer T1 of 1.05 mg/mL was selected as the optimal condition for subsequent conjugation processes. The use of borate buffer at pH 9 and incubation at 60 °C likely promoted efficient physical adsorption of aptamers onto the AuNP surface, leading to a more uniform and stable coating. This optimization not only ensures effective aptamer loading but also enhances the sensitivity and long-term stability of the biosensor system, particularly in microbial diagnostics such as *P. aeruginosa* detection (see Figure 6).

**Figure 7:**
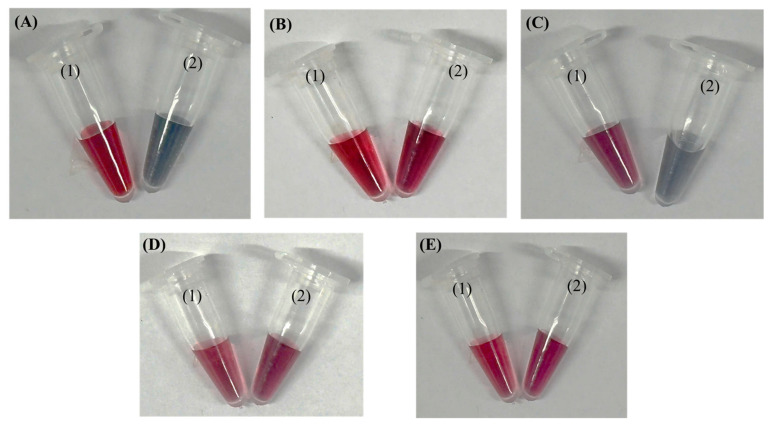
Color appearance of the solutions: (**A**) AuNPs; (**B**) AuNP–aptamer conjugates; (**C**) AuNP–aptamer conjugates incubated with *P. aeruginosa*; (**D**,**E**) AuNP–aptamer conjugates incubated with non-target bacteria (*K. pneumoniae* and *S. aureus*, respectively) before (1) and after (2) the addition of NaCl.

We also developed a simple tube-based precipitation assay to verify that the AuNP–aptamer conjugates specifically bind to target bacteria. When salt is added, the unconjugated AuNP–aptamer aggregates—turning purple/blue—whereas in wells containing bacteria, binding immobilizes the conjugates and preserves the red color of dispersed AuNPs, providing a clear visual indication of successful target recognition. This mechanism parallels previously described aptamer–AuNP colorimetric assays where target-induced aptamer detachment destabilizes nanoparticles, triggering salt-induced aggregation and color change [26,27,28,29]. Figure 7 shows the color appearance of the solutions, where 7A shows the AuNPs, 7B shows AuNP–aptamer conjugates, 7C shows AuNP–aptamer conjugates incubated in a tube with a *P. aeruginosa*, and 7D,E show AuNP–aptamer conjugates incubated with non-target bacteria (*K. pneumoniae* and *S. aureus*, respectively), before (1) and after (2) the addition of NaCl. Upon salt addition, bare AuNPs underwent pronounced aggregation, as indicated by a visible color shift from wine-red to blue–purple. In contrast, AuNPs functionalized with aptamers retained their characteristic red color, demonstrating that the aptamers stabilized the nanoparticles and prevented salt-induced aggregation. However, in the presence of the target (*Pseudomonas*), the aptamers bound specifically to the bacteria and were effectively “pulled away” from the AuNP surface, thereby losing their protective effect. As a result, the AuNPs became susceptible to aggregation upon salt addition, leading to a color change similar to that of bare AuNPs. In contrast, no color change was observed for the non-target bacterial samples.

### 3.3. Evaluation of the Hybrid LFIA Strip for P. aeruginosa Detection

#### 3.3.1. Specificity Assessment Using Blank and Non-Target Bacteria

**Figure 8:**
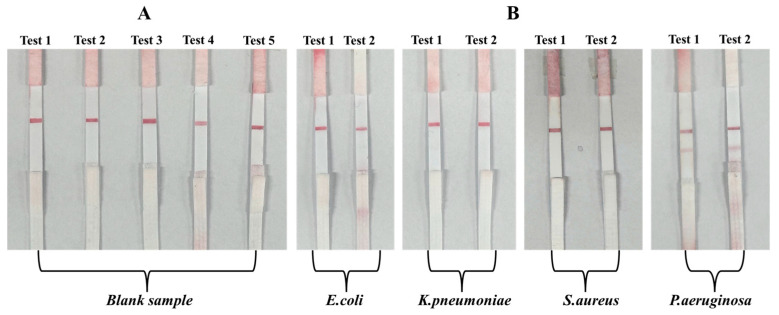
Specificity evaluation using blank, *E. coli*, *K. pneumoniae*, *S. aureus*, and *P. aeruginosa* samples: (**A**) images of test strips after testing with a blank sample (no bacteria); (**B**) images of test strips after testing with *E. coli*, *K. pneumoniae*, *S. aureus*, and *P. aeruginosa* samples, each at the same concentration of 10^3^ CFU/mL.

To evaluate the specificity of the developed hybrid LFIA strip, initial tests were conducted using blank buffer samples and suspensions of non-target bacterial strains. The blank samples served to confirm the absence of non-specific signal generation in the test zone, ensuring that the aptamer–AuNP conjugates remained stable without producing false-positive results. Non-target bacteria, including *E. coli*, *K. pneumoniae*, and *S.aureus*, were tested at concentrations of 10^3^ CFU/mL to assess potential cross-reactivity. A specific LFIA system is expected to yield a visible test line only when the target pathogen, *P. aeruginosa*, is present, while non-target strains should produce negligible or no detectable signal. The results are presented in Figure 8. The findings show that blank buffer samples and suspensions of non-target bacteria, including *E. coli*, *K. pneumoniae*, and *S. aureus*, consistently produced only a single visible C line, with no detectable signal at the T-line position. This outcome confirms that the aptamer T1–AuNP conjugates did not bind non-specifically to non-target bacterial cells, thereby preventing false-positive results. In contrast, samples spiked with *P. aeruginosa* at a representative concentration of 10^3^ CFU/mL generated two distinct lines—a clear T line alongside the C line—demonstrating the specific recognition capability of aptamer T1 for the target pathogen. The presence of a visible C line in all assays verified the proper flow and functionality of the LFIA strips, while the selective appearance of the T line only in the presence of *P. aeruginosa* confirmed the assay’s specificity.

#### 3.3.2. Sensitivity Evaluation with Spiked *P. aeruginosa* Samples

To determine the analytical sensitivity of the developed LFIA, a series of *P. aeruginosa* samples with known concentrations was prepared by spiking bacterial suspensions into the assay buffer. This approach allowed precise control of target concentrations, enabling accurate assessment of the lowest detectable bacterial load. The resulting test strips were visually examined and semi-quantitatively analyzed to establish the assay’s limit of detection (LOD) and its performance across a range of bacterial concentrations.

The performance of the LFIA strip incorporating aptamer T1 was evaluated using *P. aeruginosa* samples spiked with bacterial concentrations ranging from 10^1^ to 10^9^ CFU/mL. For each concentration, the AuNP–aptamer T1 conjugate was mixed with the sample and incubated at 60 °C for 30 min to promote target recognition and the formation of the AuNP–aptamer–bacteria complex. The resulting mixture was then applied to the sample pad of the test strip, and the results were recorded after 10–15 min.

**Figure 9:**
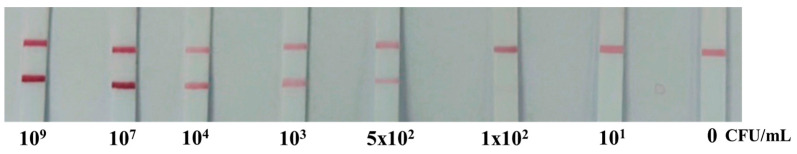
LFIA strip results across varying *P. aeruginosa* concentrations.

In positive samples containing *P. aeruginosa* at concentrations from 5 × 10^2^ to 10^9^ CFU/mL, both the control (C) and test (T) lines were visible, with the intensity of the T line varying according to bacterial concentration (Figure 9). At high concentrations (10^9^–10^7^ CFU/mL), the T line appeared intense and well-defined, indicating efficient capture of the AuNP–aptamer–bacteria complex at the test zone. As the bacterial concentration decreased to 10^4^–5 × 10^2^ CFU/mL, the T line became progressively fainter but remained visible to the naked eye. At 10^2^ CFU/mL, the T line was very faint and difficult to discern, whereas at 10^1^ CFU/mL, it was almost invisible.

**Figure 10:**
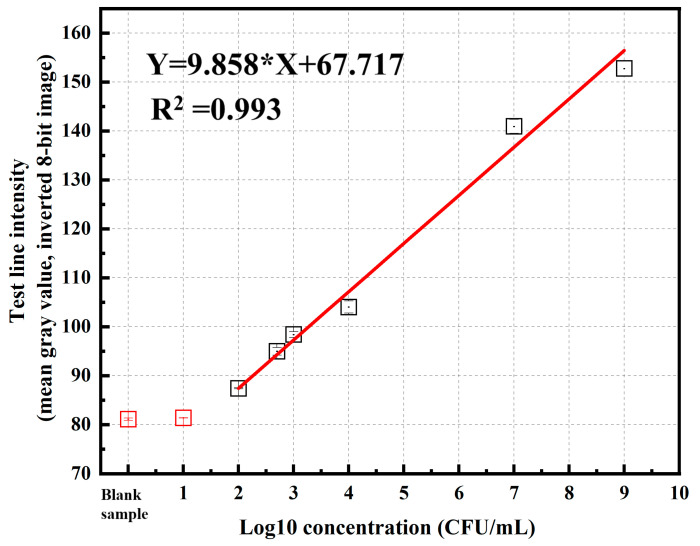
Calibration curve showing the relationship between the T-line intensity and the concentration of *P. aeruginosa* in the sample.

For semi-quantitative analysis, images of the test (T) and control (C) regions of the LFIA strips were processed using ImageJ software (https://imagej.nih.gov/ij/, accessed on 13 August 2025). ImageJ is an open-source, free image analysis tool widely used in scientific research for its ability to measure signal intensity based on pixel density with high accuracy and reproducibility. Its use standardizes the analysis process, reduces bias from visual assessment, and enables the export of digitized data for statistical processing, thereby improving the reliability of LFIA semi-quantitative results. Images were acquired in 8-bit grayscale format, where the pixel intensity values range from 0 (black) to 255 (white). This bit depth was chosen to ensure sufficient contrast and detail for accurate feature extraction, while minimizing file size and processing load. The grayscale mode also eliminates the influence of color information, focusing the analysis entirely on brightness variations. To enhance the distinction between the test line and the background, the Invert LUT mode was applied prior to measurement, making strong positive test lines appear as bright areas. This adjustment facilitated more accurate quantification of pixel intensity values for both the test and control lines. The resulting intensity data were then used to construct a calibration curve, in which the measured signal intensity was proportional to the concentration of *P. aeruginosa* detected on the LFIA strip. Table 2 and Table 3 present the T-line color intensity values of the blank sample and the *P. aeruginosa* samples at different concentrations, respectively. Based on these values, we constructed a calibration curve showing the relationship between the T-line intensity and the concentration of *P. aeruginosa* (Figure 10). The calibration curve obtained for the LFIA strip using *P. aeruginosa* spiked samples showed a strong linear relationship between the test line intensity (y) and bacterial concentration (x) within the range of 10^1^ to 10^9^ CFU/mL, described by the equation y = 9.858x + 67.717 with an excellent correlation coefficient (R^2^ = 0.993). The high R^2^ value indicates that the assay provides consistent and highly reproducible quantitative responses across the tested concentration range. The slope of the regression line (9.858) reflects the sensitivity of the assay, demonstrating a clear increase in signal intensity with increasing bacterial load. Based on this calibration curve, the LOD and LOQ were determined to be 2.34 × 10^2^ and 7.02 × 10^2^ CFU/mL, respectively, with the standard deviation of the blank signal calculated as 1.109. These results indicate that the developed LFIA strip exhibits a high analytical sensitivity, with a limit of detection as low as 2.34 × 10^2^ CFU/mL, which is lower than or comparable to many antibody-based LFIA assays reported for *P. aeruginosa*. The relatively low standard deviation of the blank signal (1.109) suggests stable baseline performance, ensuring reliable detection at low bacterial concentrations. This sensitivity is sufficient for early-stage detection in relevant clinical and environmental samples without the need for signal amplification steps.

#### 3.3.3. Demonstrating Clinical Readiness Using Simulated Positive Samples in Authentic Matrices

Due to the unavailability of ethically approved clinical samples within the timeframe of this study, direct clinical testing could not be performed. Nevertheless, the LFIA device was purposefully designed with real clinical application in mind, incorporating features to ensure compatibility with complex biological matrices. For instance, in the LFIA strip for *P. aeruginosa* detection, we integrated an FR1-blood separation membrane (MDI membrane technology) into the sample pad. In a whole-blood detection configuration, this membrane plays a critical role in removing red blood cells. Its microporous structure, high void volume, and strong retention capacity enable selective and efficient removal of cellular components—particularly erythrocytes—while allowing plasma and target analytes such as intact bacteria or bacterial surface molecules (e.g., LPS, membrane antigens) to pass through. Minimal analyte adsorption within the membrane further enhances strip sensitivity. This integrated design provides a strong basis for direct evaluation with whole-blood clinical samples in the next research phase.

**Figure 11:**
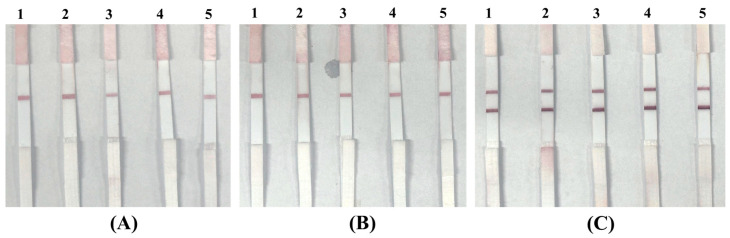
LFIA strip results for IBV-positive field samples and simulated positive samples: (**A**) five unmodified IBV-positive field samples (negative for *P. aeruginosa*); (**B**) IBV-positive samples spiked with *S. aureus* (10^9^ CFU/mL, non-target); (**C**) IBV-positive samples spiked with *P. aeruginosa* (10^9^ CFU/mL, target).

To further demonstrate the LFIA system’s clinical readiness in the absence of approved human clinical samples, we employed real biological matrices from a parallel project in our group. These matrices were obtained from poultry infected with the infectious bronchitis virus (IBV) and represented authentic, complex sample backgrounds (see Table 4). Using these matrices, we prepared three groups of simulated samples: (i) IBV-containing samples spiked with *P. aeruginosa* (target bacterium); (ii) IBV-containing samples spiked with *Staphylococcus aureus* (Gram-positive, non-target bacterium); and (iii) IBV-containing samples without *P. aeruginosa* (negative control). The test results are presented in Figure 11, where Figure 11A shows the strip images for five unmodified IBV-positive field samples; Figure 11B shows the strip images for IBV-positive samples spiked with *S. aureus* (10^9^ CFU/mL); and Figure 11C shows the strip images for IBV-positive samples spiked with *P. aeruginosa* (10^9^ CFU/mL). A clear test line (T line) was observed only in the *P. aeruginosa*-spiked samples, confirming specific detection. In addition, our LFIA design employs two types of conjugates: (i) IgG–AuNP conjugates captured at the control (C) line to verify assay validity; and (ii) aptamer–AuNP conjugates specific to *P. aeruginosa* for target capture at the T line. In negative matrices (IBV-positive or spiked with *S. aureus*), only the C line appeared, with most conjugates migrating fully to the absorbent pad. In positive matrices, both C and T lines showed comparable intensities, and the absorbent pad remained clear, indicating complete capture of aptamer–conjugates at the T line. These findings demonstrate that our LFIA strip remains stable, sensitive, and highly specific even in complex biological matrices, underscoring its clinical readiness and robustness for future direct testing with whole-blood clinical samples.

### 3.4. Advantages, Limitations, and Perspectives of Aptamer-Based LFIA

In this study, a modified whole-cell SELEX approach was implemented using inactivated *P. aeruginosa* cells, with counter-selection against *E. coli* and *K. pneumoniae* in the fifth and seventh rounds to improve specificity [30]. After 10 rounds of selection, six aptamers with high binding affinity were identified, with T1 exhibiting stable structures and superior binding performance. Aptamer T1 was integrated into an LFIA system, replacing the conventional detection antibody. The detection mechanism involved AuNP–aptamer complexes that bind to target bacteria and are captured at the test line (T) by immobilized antibodies, producing a red signal. Experimental results showed that T1 aptamers specifically detected *P. aeruginosa* with a limit of detection of 2.34 × 10^2^ CFU/mL within 15 min. The developed LFIA offers rapid response, low cost, ease of use, and no requirement for specialized equipment, making it suitable for POCT, especially in resource-limited settings.

To better contextualize the performance and applicability of our proposed point-of-care (POC) detection system, we compared it with other reported lateral flow immunoassay (LFIA) platforms for Pseudomonas detection. Table 5 summarizes the main analytical features, target types, detection limits, assay times, and detection principles of representative systems. This comparison highlights the distinct advantages of our approach in terms of simplicity, rapidity, and field applicability, particularly for on site testing without the need for specialized laboratory infrastructure.

A notable limitation of the hybrid LFIA using aptamers instead of antibodies is the requirement for a minimum incubation time of 30 min to ensure efficient binding of the aptamer to the target bacteria. Therefore, the aptamer–gold nanoparticle conjugates cannot be stored on the conjugate pad as in conventional LFIA but must be preserved in solution form and incubated separately with the sample before running the test strip. This reduces user convenience, especially in rapid on site diagnostics. To overcome this limitation and enhance the practical applicability, several improvements could be implemented in future research. First, optimizing the aptamer structure through advanced SELEX approaches could shorten the binding time to bacteria, thereby reducing the required incubation time. Second, redesigning the physical structure of the test strip—for example, integrating a pre-loaded conjugate chamber containing the freeze-dried aptamer–AuNP along with Mg^2+^/Na^+^ salts, BSA, trehalose, and PEG/dextran as crowding agents for rapid rehydration and increased on-rate—could simplify the user workflow without compromising sensitivity and specificity. Third, employing a paper-folding (origami LF) format to create a closed incubation chamber for aptamer–AuNP with the sample, which initiates flow only after a preset incubation time when the user folds the strip, could further streamline the process. These innovations would not only improve the performance of aptamer-based LFIA systems but also expand their applicability in rapid point-of-care diagnostics.

## 4. Conclusions

This study established an improved whole-cell SELEX protocol using inactivated *Pseudomonas aeruginosa* cells, coupled with in silico screening based on thermodynamic stability and molecular docking analysis. Six high-affinity aptamers were identified, among which T1 exhibited superior structural stability and binding performance. The aptamer T1 was successfully conjugated to gold nanoparticles and integrated into a hybrid lateral flow immunoassay (LFIA), replacing conventional detection antibodies. The resulting platform enabled rapid, visual detection of *P. aeruginosa* with high specificity and a detection limit as low as 2.34 × 10^2^ CFU/mL, without requiring specialized instrumentation.

The methodology offers broad adaptability for future diagnostic applications. Whole-cell SELEX enables aptamer selection directly against intact microbial cells, eliminating the need for purified antigens and ensuring recognition of native surface epitopes. The hybrid LFIA format—combining aptamer-based detection with antibody-mediated capture—provides a robust, flexible architecture that can be readily adapted to other bacterial or viral targets simply by substituting the aptamer component.

Given their high chemical stability, low production cost, and strong specificity, aptamers represent a promising alternative to antibodies in diagnostic assays. The platform developed here serves as a proof-of-concept for a new generation of aptamer-based diagnostics that are rapid, scalable, and suitable for point-of-care testing, particularly in resource-limited settings. Future work will focus on extending this approach to clinically and epidemiologically important pathogens, such as Salmonella spp., Mycobacterium tuberculosis, and multidrug-resistant Acinetobacter baumannii, thereby contributing to improved infectious disease surveillance, outbreak preparedness, and global health response.

## Figures and Tables

**Table 1:** Characteristics of the six selected aptamers (including the nucleotide sequences, docking score, predicted minimum free energy (ΔG), frequency (%), diversity score, and the corresponding two-dimensional (2D) and three-dimensional (3D) structural and molecular docking (MD) configurations based on the MFE model of the six selected aptamers).

Aptamer Name	Aptamer DNA Sequence	Docking Score	MFE (kcal/mol)	Frequency (%)	Diversity Score	Predicted Secondary Structure (2D) Based on MFE Model	Predicted Tertiary Structure (3D) Based on MFE Model	Molecular Docking with CPA
T1	ATCCGTCACACCTGCTCTGTAAACACCTACGGTCTTAGCATACGGTATAAGCCGTAACCGGTTTTACCTAAACTGGTGTTGGCTCCCGTAT	−453.85	−27.10	24.98	4.64	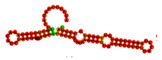	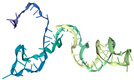	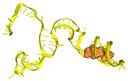
T2	ATCCGTCACACCTGCTCTCAAAGGTCATAGGGGCTTCTTGGCAGCGTAATGCCTTGCTCGCATTTTTCCCTCATGGTGTTGGCTCCCGTAT	−366.23	−22.11	3.82	28.74	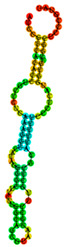	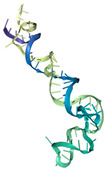	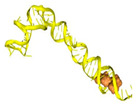
T3	ATCCGTCACACCTGCTCTCATCATGCCCGCGTTCTAATAACTGCTATATCCTTTATCGCCTCTATCCCTCCGTTGGTGTTGGCTCCCGTAT	−409.72	−12.20	8.83	13.74	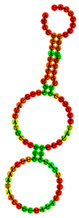	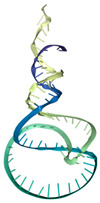	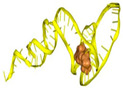
T4	ATCCGTCACACCTGCTCTGAGACTAGCAGTTTTTAACCAGAGTAAATAACTCCCCTCTTCCTAAAATTTCCCCTGGTGTTGGCTCCCGTAT	−427.08	−15.86	21.23	8.54	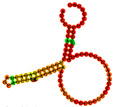	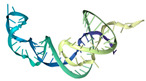	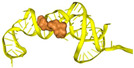
T5	ATCCGTCACACCTGCTCTCGGTGGTCAGCATCTCACTTGCCTTCTGTCCTGACCTATCCATCCCTCGTCGTCATGGTGTTGGCTCCCGTAT	−373.65	−18.11	1.15	19.52	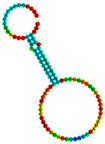	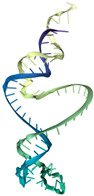	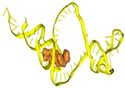
T6	ATCCGTCACACCTGCTCTCAGTATACACCCGTTCTCCGTCTGGTCTACAGTCCCCCGGCTGAGCCCCTATTCGTGGTGTTGGCTCCCGTAT	−378.06	−18.83	5.12	22.07	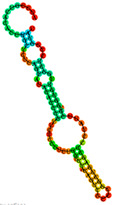	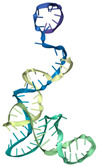	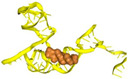

Note: structures were predicted using RNAfold (for 2D) and RNAComposer (for 3D) based on the MFE model.

**Table 2:** T-line color intensity values for the blank samples.

Blank Sample	Test Line Intensity (Mean Gray Value, Inverted 8-Bit Image) (a.u.)	Mean Value	STDEV
1	81.029	80.771	1.109
2	79.317		
3	79.656		
4	79.456		
5	81.370		
6	81.357		
7	82.507		
8	81.368		
9	81.024		
10	81.924		

**Table 3:** T-line color intensity values for *P. aeruginosa* samples at different concentrations.

*P. aeruginosa* Concentration (CFU/mL)	Log10 Value		Test-Line Intensity (Mean Gray Value, Inverted 8-Bit Image) (a.u.)	Mean Value	STDEV
10^9^	9.00	Test 1	154.499	152.766	1.609
		Test 2	151.320		
		Test 3	152.479		
10^7^	7.00	Test 1	139.736	140.934	1.428
		Test 2	140.552		
		Test 3	142.515		
10^4^	4.00	Test 1	103.569	104.022	1.239
		Test 2	105.424		
		Test 3	103.074		
10^3^	3.00	Test 1	98.232	98.383	0.604
		Test 2	99.048		
		Test 3	97.887		
5 × 10^2^	2.70	Test 1	95.887	94.974	0.792
		Test 2	94.569		
		Test 3	94.468		
10^2^	2.00	Test 1	87.526	87.405	0.106
		Test 2	87.331		
		Test 3	87.357		
10^1^	1.00	Test 1	81.368	81.370	0.014
		Test 2	81.357		
		Test 3	81.370		
Blank sample	0.00	Test 1	81.357	81.102	0.227
		Test 2	81.024		
		Test 3	81.940		

Abbreviation: STDEV standard deviation.

**Table 4:** Collected poultry samples and IBV detection results by real-time PCR.

No.	Code	Type of Sample	Province /City	IBV-Positive Sample Confirmed by Real-Time PCR	Ct Value
1	230713.2	Feces swab	Bắc Ninh	positive	25.75
2	230716.1	Mouth swab	Bắc Ninh	positive	27.66
3	240227.1	Oral swab	Lai Châu	positive	30.00
4	240227.2	Rectal swab	Lai Châu	positive	24.50
5	240228.1	Oral swab	Gia Lai	positive	35.20

**Table 5:** Comparison of reported LFIA-based methods for *Pseudomonas* detection with the present hybrid LFIA system.

Method	Signal Type	LOD	Assay Time	Sample Type	Advantages	Ref
RPA-LFS LasB gene	AuNP Colorimetric	10^3^ CFU/mL	1 h	DNA from bacteria (culture/clinical)	High sensitivity and specificity, no PCR instrument required	[31]
MCDA-LFB (MCDA+AuNP-LFA)	AuNP Colorimetric	10 fg DNA	40 min	DNA from bacteria	Rapid, visual, no instrument required	[32]
Mag@QDs-WGA LFA	Fluorescent LFA	10^1^–10^2^ CFU/mL	35 min	Food/environmental samples	Very high sensitivity, multiplexing capability, broad-spectrum	[33]
LAMP-LFB (ophthalmic *P. aeruginosa*)	AuNP Colorimetric	10^2^ CFU/mL	1 h	Biological DNA samples	Simple, suitable for clinical use	[34]
Hybrid AuNP–aptamer + antibody LFIA	AuNP Colorimetric	10^2^ CFU/mL	15 min	Whole bacterial cells	Sensitive, rapid, simple operation at point-of-care	This work

## Data Availability

The original contributions presented in this study are included in the article. Further inquiries can be directed to the corresponding author.
